# The State and the Osteopaths

**Published:** 1905-03

**Authors:** 


### The State and the Osteopaths.

THROUGH the courtesy of Dr. Frank Van Fleet, the efficient chairman of the committee on legislation of the Medical Society of the State of New York, we have received a copy of senate bill, No. 298, introduced by Senator Davis, which is entitled, an act regulating the practice of osteopathy. This bill would create an additional schism in the practice of medicine, and give the state the confusion and complication incidental to five sets of state and county medical societies where there should be but one. The bill is informing and should be conclusive as to the nature of osteopathy, and from its provisions we are told that "osteopathy means that science or system of healing which treats diseases of the human body by manual therapeutics;" and, further, that "osteopathists when duly licensed and registered in accordance with this act shall have all the rights and privileges and be subject to the rules and regulations that govern other physicians."

This is in exact harmony with the facts in the case, and corresponds with the ideas we have had upon the subject for years; moreover, it seems to establish the fact, at least, so far as the osteopaths are able to do so, that osteopathy is the practice of medicine and that the osteopaths have been practising medicine, are practising medicine and intend to practise medicine.

The vital point of this provision is that "diseases of the human body" are to be treated. The public may need to be told that "manual therapeutics" is not unknown to the profession of medicine,—is not a discovery of the osteopaths,—but has been developed and used in medicine for centuries, and, besides, it is a most useful means of cure; but as the whole is greater than any of its parts, so therapeutics is greater and more effective than "manual therapeutics," hence the attempt to make "manual therapeutics a complete system of curing "diseases of the human body" is either ignorant or fraudulent, one or both.

Fatal defects in this bill appear in its provisions as to the qualifications of the members of the proposed state board of examiners, and as to the persons the bill would license to practise medicine without examination.

As to the members of the state board of examiners, it is provided that they shall be reputable graduates of a regularly conducted school of osteopathy and shall have practised osteopathy in this state for at least one year.

It is to be noted of those provisions that the questions of who is a reputable graduate and which school is regularly conducted, are left to the decision of the osteopaths themselves, the state having no opportunity of determining what was the preliminary educational equipment of the candidate, of what advantage he took of the teachings of his school, or of what were the educational standards of that school.

It is also to be noted that the test of good moral character is not to be applied to any of these candidates for examination; for, interpreted in the light of the medical practice Act, they have been in daily violation thereof, and just how this criminal life is compatible with good moral character might be difficult to establish. An essential defect of the bill, in our judgment, is its failure to exact the usual educational qualifications, preliminary and professional, of those about to be licensed to practise medicine. The bill would license "any person who at the time of the passage of this act shall be actually engaged in the practice of osteopathy in this state, and who is a reputable graduate of a regularly conducted school of osteopathy."

Here is a wholesale licensing of persons to the practice of medicine without one iota of protection as to preliminary education, term of professional study, advantage actually derived from such professional training, or as to good moral character. It seems incredible that the state of New York could be pursuaded to place upon its records a law bearing so absurd a proposition as this—

namely, that neither preliminary education, professional preparation nor moral character shall be required of those who are to assume the responsibility of the practice of medicine.

The provisions of the bill as to future candidates for admission to the practice of medicine through this gateway,—door number five,—make the bill exceedingly dangerous from an *apparent* willingness to meet existing standards as to preliminary education, length of courses in a given year, number of years of study, subjects for examination by state examiners, good moral character and other essentials. It would seem that the bill is drawn to make it easy for the osteopaths now at work to become doctors and also provides restrictions sufficient to keep down future competition.

It is difficult to regard with seriousness the proposition to make the control of the practice of massage a part of the educational scheme of the University of the State of New York.

We believe the bill to be dangerous in general and in particular and we warn our readers that nothing short of vigorous and united opposition will secure its defeat.

